# The Exosome Journey: From Biogenesis to Regulation and Function in Cancers

**DOI:** 10.1155/2022/9356807

**Published:** 2022-07-18

**Authors:** Yuan Li, Li Meng, Bo Li, Yanxia Li, Tao Shen, Baobing Zhao

**Affiliations:** ^1^Key Lab of Chemical Biology (MOE), School of Pharmaceutical Sciences, Cheeloo College of Medicine, Shandong University, Jinan 250012, Shandong, China; ^2^NMPA Key Laboratory for Technology Research and Evaluation of Drug Products, School of Pharmaceutical Sciences, Cheeloo College of Medicine, Shandong University, Jinan 250012, Shandong, China; ^3^Suzhou Research Institute, Shandong University, Suzhou 215123, Jiangsu, China; ^4^Department of Orthopedics, Sun Yat-Sen Memorial Hospital of Sun Yat-Sen University, Guangzhou 510000, China

## Abstract

Exosomes are a type of small endosomal-derived vesicles ranging from 30 to 150 nm, which can serve as functional mediators in cell-to-cell communication and various physiological and pathological processes. In recent years, exosomes have emerged as crucial mediators of intracellular communication among tumor cells, immune cells, and stromal cells, which can shuttle bioactive molecules, such as proteins, lipids, RNA, and DNA. Exosomes exhibit the high bioavailability, biological stability, targeting specificity, low toxicity, and immune characteristics, suggesting their potentials in the diagnosis and treatment of cancers. They can be applied as an effective tool in the diagnostics, therapeutics, and drug delivery in cancers. This review summarizes the regulation and functions of exosomes in various cancers to augment our understanding of exosomes, which paves the way for parallel advancements in the therapeutic approach of cancers. In this review, we also discuss the challenges and prospects for clinical application of exosome-based diagnostics and therapeutics for cancers.

## 1. Introduction

Extracellular vesicles (EVs) are a collective term containing various types of cell-released membranous structures, including exosomes, microvesicles, microparticles, ectosomes, oncosomes, and apoptotic bodies [1, 2]. Among these EVs, exosomes are able to escape from the phagocyte system because of their small size and exert their superiority in intercellular communications. Exosomes are a type of endocytic and heterogeneous membrane-derived vesicles, which could be secreted by multiple cell types to induce or inhibit different cellular and molecular pathways, including immune cells, stem cells, and tumor cells [3, 4]. Exosomes used to be regarded as garbage cans for abandoning redundant cell materials. Recently, the roles of exosomes are gradually suggested, including immune regulation, intercellular communications, and biological events.

Cancer is the second leading cause of death all over the world [5]. Continuous improvements in the treatment of cancers, including surgery, chemotherapy, and radiotherapy, have significantly increased the survival, but these strategies cannot effectively control the recurrence and metastasis of cancers [6]. Thus, novel therapeutic methods are urgently needed. Since exosomes can play key roles in various physiological and pathological processes, it seems likely that they can also affect a variety of pathophysiological processes of cancers, including cancer development, migration, drug resistance, and metastasis. In this review, we comprehensively searched the PubMed database (https://pubmed.ncbi.nlm.nih.gov/) with the combined keywords “exosomes” and “cancers,” and focus on the biogenesis, regulation, and function of exosomes in cancers, which are capable of being used as a powerful weapon for the treatment of cancers.

## 2. Exosomes

### 2.1. Characteristics and Biogenesis

In 1981, Trams and his colleagues firstly described exosomes as microvesicles, which contained 5′-nucleotidase activity, released by neoplastic cell lines [7]. However, after that, the concept had been corrected and the endocytic origins of exosomes were proven, which had been regarded as natural nanocarriers. Exosomes are characterized by having a size between 30 and 150 nm, a cup-shaped structure, a density of 1.13–1.19 g/mL, and unique double lipid layer [8–10]. In addition, exosomes exhibit high biocompatibility and low toxicity. Exosomes are considered as a crucial player in the intercellular communication by transferring cellular contents to recipient cells [11]. They could transfer the bioactive molecules from cancer cells to cells in tumor microenvironment, thereby facilitating the development and progression of cancers [12]. The bioactive molecules in exosomes are transferred to nearby cells or recipient cells faraway, where they can modulate the signaling pathways involved in the cell proliferation, differentiation, and apoptosis. Exosomes regulate the biological behaviors of cancer cells, tumor microenvironmental cells, and distant recipient cells in this way and participate in the growth, invasion, metastasis, angiogenesis, and drug resistance of cancer cells [13].

Exosomes are commonly obtained from cell culture supernatants and various body fluids, including blood, cerebrospinal fluid, saliva, breast milk, bile, and urine. Exosomes contain a multitude of biomolecules, such as DNA, RNA, mRNA, lipids, metabolites, cytosolic, and proteins, and play essential roles in intercellular communications [14]. They can be secreted after fusion of multivesicular endosomes with the cell surface or directly sprout from plasma membranes. The biogenesis of exosomes mainly includes four stages, including initiation (membrane formation), endocytosis, formation of multivesicular bodies (MVBs), and sorting (secretion/degradation/recycling) [11]. At first, the cell membrane with ubiquitinated surface receptors is internalized endocytosis of the plasma membrane. Then, endosomal sorting complexes, which are necessary for the transport-0 (ESCRT-0), can bind to the ubiquitinated surface receptors via a FYVE domain to be changed into the early endosomes. And MVBs are generated in the form of trans-budding when ESCRT-I and ESCRT-II are assembled into the endosome membranes. At last, ESCRT-III induces the release of exosomes by fusing with the plasma membrane or the degradation by fusing with lysosomes.

### 2.2. Isolation and Purification

Differential ultracentrifugation method is usually used for the isolation of exosomes. The exosomes were extracted and isolated through multiple centrifugation steps, containing increasing centrifugal strength to sequentially pellet cells, microvesicles, and exosomes [15]. The method is fast and simple, while soluble proteins and nonexosomal particles in exosomes are hardly removed. Based on the centrifugation, ExoQuick precipitation method is also applied to isolate exosomes. After chemically precipitated with ExoPrep solution, the ultracentrifugation is performed. The advantages and disadvantages are similar to the differential ultracentrifugation method. Besides, there are also other techniques, such as ultrafiltration, antibody-coated magnetic beads, commercial precipitation kits, microfiltration techniques, and the state-of-the-art microfluidic technology, used for exosome separation.

Until now, the purest exosomes are isolated through density gradient isolation method by using sucrose or iodixanol. This method can obtain the purest exosomes, but are more time-consuming and more costly. Besides, microfluidic immunoisolation is applied for purification of exosomes by using antibodies against exosomal markers, such as EpCAM and CD63. This method is centered on exosomes expressing the target surface markers rather than other subpopulations of exosomes (27440105). There are also other novel detection modalities, such as biosensing and basic proteomics methods. Despite their unique characteristics, there is still a long way in the research of exosome-based assays.

### 2.3. Identification

Accurately detecting exosomes are important in the biology of vesicles. Currently, optical techniques and nonoptical methods are applied to characterize the vesicles. The optical methods contain nanoparticle tracking analysis (NTA), dynamic light scattering (DLS), fluorescence signals, and flow cytometry. The nonoptical methods include resistive pulse sensing (RPS), micronuclear magnetic resonance (*μ*NMR), nanoholographic imaging, atomic force microscopy (AFM), enzyme-linked immunosorbent assay (ELISA), transmission electron microscopy (TEM), field-effect transistors (FET), lateral flow immunoassay (LFIA), and Raman spectroscopy. The above and emerging exosome-based technologies are helpful and urgently needed for the application of exosomes in the diagnosis and treatment of cancers. With gradually better understanding of exosomes, the corresponding technologies are also improving, which is helpful for developing the exosome-based application.

## 3. Exosomal RNAs in Cancers

Exosomes comprise various cellular elements, which are reflected by parental cells and affect the development of cancers. Increasing evidence shows that the functions of exosomes in cancers depend on their cargos and the identification of the components is one of the major challenges in the fields [16]. The uniqueness and enrichment of exosomal nucleic acids (RNAs) have been recently reported to be produced during the process of endosomes endocytose, collecting a large number of noncoding sequences. There are mainly three types of RNAs, including microRNAs (miRNAs), long noncoding RNAs (lncRNAs), circular RNAs (circRNAs), and other noncoding RNAs (ncRNAs) [17, 18].

### 3.1. miRNAs

It is widely accepted that miRNAs are a class of evolutionarily endogenous small noncoding RNAs that are about 20 to 25 nucleotide long sequences at length, which can bind to the target mRNAs and negatively regulate the gene expression via post-transcriptional inhibition or target mRNA degradation [19, 20]. As reported that, miRNAs are involved in the development and progression of cancers, which have brought increasing attraction [21]. For example, prostate cancer (PCa) cell-derived exosomes promote the angiogenesis, while the exosomes obtained from noncancer individuals are regarded as suppressors in the local environment [22]. Owing to the specific sorting mechanisms, exosomes contain greater concentrations of miRNAs, which are more stable than circulating miRNAs. The stability of miRNAs in exosomes is significantly correlated with the expression levels, which are able to transfer the information to various cellular processes [23]. According to the results of a large cohort including 195 non-small-cell lung cancer (NSCLC) patients and 30 healthy controls, in the research by Dejima et al., the plasma levels of exosomal miR-21 and miR-4257 derived from NSCLC were markedly higher in patients with recurrence than that in patients without recurrence or healthy individuals [24]. Besides, this study also indicated that the higher levels of exosomal miR-21 or miR-4257 are correlated with a worse prognosis with a shorter disease-free survival (DFS) [24]. It has been demonstrated that hepatocellular carcinoma (HCC) cells (Huh7)-derived exosomes restrain the growth and promote the aging of HepG2 cells by transferring miR-122 [25]. Previous studies indicated that breast cancer cell-derived exosomes promoted the growth, metastasis, and autophagy of breast cancer cells by transferring miR-1910-3p [26]. It has been shown that exosome-derived miR-200b promotes the proliferation of colorectal cancer, which is transferred to the recipient cells and suppresses the expression of p27 in the target cells [27]. Growing body of evidence indicated that exosomal RNAs play important roles in cancers (Table 1). With the in-depth research on the transportation functions of exosomes, exosomal miRNAs obtain a focus of attention. Thus, miRNAs play crucial roles in the development and progression of cancers.

### 3.2. lncRNAs

Other molecules, such as lncRNAs, have also been found in exosomes. LncRNAs contain 200 nucleotide sequences at length, which can regulate the expression of genes or proteins via coordinating epigenetic, transcriptional, and post-transcriptional steps [69]. Numerous studies showed that lncRNAs exert their functions via multiple molecular mechanisms, including binding with DNA to regulate the gene transcription, acting as the competing endogenous RNA (ceRNA) to control the gene expression at post-transcriptional level, combining with proteins, and encoding functional small peptides [70]. Interestingly, lncRNAs could be selectively sorted from exosomes and involved in the intercellular communication in the tumor microenvironment [71].

Exosomal lncRNAs can play important roles in the occurrence and development of cancers, including proliferation, invasion, angiogenesis, and drug resistance, which might act as attractive therapeutic targets and diagnostic biomarkers [72] (Table 2). A recent study showed that in patients with NSCLC, the exosomal lncRNA MALAT-1 was much higher than healthy controls. Besides, the upregulation of exosomal lncRNA MALAT-1 is closely related to an advanced tumor stage and lymphatic metastasis [84]. Although a growing number of evidence reveals that found that lncRNAs could be transferred between various cells, which is related to exosomes, many questions are still doubtful. For example, it is unclear whether nuclear and cytoplasmic lncRNAs exert the similar function that can be loaded into exosomes, and how their sequences, structures, and protein-binding partners affect the sorting. How many copies of lncRNAs are involved in exosomes are needed for phenotypically affecting the recipient cells? There is a dramatical difference between physiological exosome communication and in vitro treatment with purified vesicle preparations in these aspects, including time and intensity, which require further investigation and careful evaluation. In addition, while lncRNAs are upregulated in specific cancers in comparison with normal tissues and could be detected in exosomes in the circulation of patients with cancers, it is unclear whether the incorporation into exosomes depends on the specific sorting mechanisms in cancer cells [86].

### 3.3. circRNAs

As a type of exosome-derived noncoding RNAs, exosomal circRNAs have been found to play key roles in the development and progression of cancers and act as novel diagnostic and prognostic biomarkers in cancers. CircRNAs are a class of tissue specific and covalently closed circular noncoding RNA, which is widely present in eukaryote. Exosomal circRNAs are novel frontiers in cancer research, and the important exosomal circRNAs are summarized in Table 3. These studies have suggested that exosomal circRNAs might exhibit an important influence on the development and occurrence of cancers, which suggested the potential diagnostic and therapeutic values of exosomal circRNAs. To explore the mysterious connections of exosomes and circRNAs, it might provide a key hint to probe into the biological functions of exosomal circRNAs. In addition, studying the pathogenesis mechanisms of cancers and identifying novel promising diagnostic biomarkers and therapeutic targets are a popular topic in the future.

## 4. Exosomal Proteins in Cancers

Exosomes can carry a broad variety of molecules depending on the origins and in vitro culture conditions, such as proteins. It has been shown that exosomes can express a wide range of proteins involved in membrane-related functions, including Rab GTPase, Annexin, cellular adhesion proteins (integrins and tetraspanins), cytoskeletal proteins (actin and myosin), and heat shock proteins (Hsp70) [112]. Recent studies suggest that membrane transport and fusion proteins in exosomes play vital roles in the occurrence and development of cancers, including annexins, RAB5/RAB7, and TSG101. For example, dendritic cells (DCs)-derived exosomes are capable of carrying MHC-I, which can bind to the tumor-derived peptides, and then, the complex activates the immune cells to play crucial antitumor roles [113]. An interesting study has revealed that membrane surface protein TRAIL from exosomes can transfer the apoptosis-related signals to the tumor cells and induce the apoptosis of tumor cells [114]. Moreover, exosomes with SIRP*α* are capable of binding to CD47 on tumor cells, and promoting the phagocytosis of macrophages and finally inhibiting the cancer progression [115].

In addition to the RNAs and DNA present in exosomes, exosomal proteins could also be used as promising biomarkers (Table 4). It has been reported that exosomal EGFR protein can be regarded as a potential biomarker for the characterization of lung cancer [141, 142]. As demonstrated, the diagnostic potentials of 49 exosomal membrane-attached proteins in 336 patients with lung cancer and 127 controls are confirmed. Among these proteins, CD151, CD171, and tetraspanin 8 are reported to be the strongest biomarkers for patients with lung cancer [143].

## 5. Exosome-Based Diagnostics and Therapeutics in Cancers

### 5.1. Diagnosis

With the aging and growth of the population, the incidence and mortality of cancers are rapidly growing and cancer is one of the most leading causes of death. Cancers seriously endanger human health, and early diagnosis of cancers is required for increasing the survival rates of patients and reducing the mortality. Recently, one of the latest concepts regarding early diagnosis is based on the extracellular vesicles released by cells where the exosomes are at the frontiers. The biomarker potentials of exosomes in liquid biopsies hold huge promise and might revolutionize the diagnostics strategies of cancers. For example, exosome-derived miRNAs can act as novel potential biomarkers in the diagnostics and prediction of colorectal cancer [144, 145]. It has been found that miR-423-5p is increased in the serum exosomes of patients with gastric cancer (GC), and the levels of miR-423-5p are related to lymph node metastasis and poor outcome [146]. And the levels of miR-126 and let-7a are much higher in exosomes collected from the bronchoalveolar fluid of patients with lung adenocarcinoma than healthy controls [147]. Moreover, patients with pancreatic cancer (PC) exhibit higher level of exosomal miR-191, miR-21, and miR-451a than healthy subjects [148]. Exosomal lncRNA SAP30L-AS1 has been discovered to upregulated in benign prostatic hyperplasia (BPH) and lncRNA SChLAP1 is increased in prostate cancer than in BPH and healthy controls, which is confirmed to possess diagnostic values in distinguishing prostate cancer by the receiver operating characteristic curve [149]. A study containing 246 subjects (126 patients and 120 healthy controls) showed that the expression levels of exosomal lncRNA HOTTIP were much higher in gastric cancer patients than normal controls, which was in close correlation with invasion depth [82].

In addition, the protein levels in exosomes might also act as biomarkers in the diagnosis of cancers. As previously described, the patients with acute myeloid leukemia (AML) exhibit higher levels of plasma exosomes with a specific phenotype containing transforming growth factor beta 1 (TGF*β*-1), which has a suppressive effect in the cytotoxic activity of NK cells [150]. According to the results of proteomics analysis by mass spectrometry of urinary exosomes obtained from 16 patients with prostate cancer (PC) and 15 controls, the transmembrane protein 256 is the highest sensitivity of 94% and distinctly enriched with high specificity in patients with PC [151]. As previous detailed, an inhibitor of apoptosis protein (Survivin-2B) has been found to be differentially expressed in exosomes from patients with breast cancer, suggesting its values in the diagnosis and prognosis of breast cancer [152]. The results of gene microarray from blood exosomes of mice with glioblastoma multiforme (GBM) revealed that the levels of DNM3, p65, and CD117 are significantly increased, whereas PTEN and p53 are decreased, which might be served as novel diagnostic markers for GBM [153].

Some studies also focus on the roles of circRNAs in cancer diagnosis. For example, the level of circ-PDE8A has been detected in the plasma exosomes from patients with pancreatic ductal adenocarcinoma (PDAC) and exosomal circ-PDE8A has been found to be related to the progression and prognosis in PDAC patients [109]. Many studies have stressed the roles of exosomes in the diagnosis and prediction of cancers owing to the exosomal molecules, including miRNAs, proteins, and lncRNAs. We believe that in-depth understanding of exosomes in the diagnosis of cancers could help to design novel cancer-diagnostic and cancer-prognostic tools. Effective diagnostic strategies for cancers based on exosomes are expected in the near future. Despite these advances in exosomes-based diagnostics and prediction of cancers, there are still many challenges needed to be overcome until clinical application. Much research needs to be done about the roles of exosomes in the diagnosis and prediction of cancers in the future.

### 5.2. Treatment

Regarded as the best-studied natural nanomaterials in the past decades in cancer therapy, exosomes are able to pass through the biological barriers, such as the blood-brain barrier (BBB) and blood-tumor barrier, indicating their potentials in the diagnosis and treatment of brain cancers. Mesenchymal stem cells (MSCs)-derived exosomes have been reported to be applied in the treatment of pancreatic cancer (PC) in animal experiments by delivering KRAS, G12D, and siRNAs [154, 155]. Besides, exosomes could be applied as novel antitumor vaccines. For example, exosomes obtained from dendritic cells can deliver the melanoma antigen identified by T cells 1 (MART-1) into patients with NSCLC at IIIB/IV stage and then inhibit the progression of NSCLC and improve the survival through activation of immune response, which is revealed by phase II clinical trials [113].

Many targets of anticancer chemical agents are intracellular, which need to cross the cell membranes and then exert anticancer functions. Because of their poor water solubility and easy degradation, the chemical agents are difficult to produce the desired effects or avoid adverse reactions. Exosomes have the advantages of low immune prototype and weak side effects, whose lipid bilayer can play protective roles in the contents in vivo [156]. Besides, exosomes are easy to enter the cells via the interaction between their membrane proteins and target cells. Thus, exosomes are regarded as the best natural carriers for chemical agents [115]. Hence, a better understanding of how to engineer and deliver exosomes to specific cells is crucial to improve the cancer therapy potential of exosomes. For example, plant-derived exosomes are easily to be absorbed by the intestine and are used for inhibiting the development of colon cancer by delivering curcumin, confirmed by clinical trials (ClinicalTrials.gov identifier: NCT01294072). It has been confirmed that the treatment with DCs-derived exosomes carrying doxorubicin significantly inhibits the growth of breast cancer cells and exerts no toxicity effects on mouse models [157]. In the study of K. Tang et al., exosomes loaded with cisplatin exert significant anticancer effects on ovarian cancer and cause no side effects on other tissues in mice, including liver and kidney. And this is an advantage in comparison with cisplatin treated alone [158]. Moreover, exosomes from plants can also be used in the adjuvant therapy for cancers. It has been shown that during the treatment of head and neck cancer, the patients suffer from oral mucositis. And grape-derived exosomes are able to ease the oral mucositis and pain, revealed by clinical trials (ClinicalTrials.gov identifier: NCT01668849). Exosomes can extend the circulation half-life and block the drug accumulation in nontarget organs by targeting specific cells. Thus, the release of exosomes in biofluids could have major advantages for exploration of the complex mechanisms of tumor progression and treatment response.

To successfully develop novel and efficient therapeutic strategies in the treatment of cancers, exosomes should not be disregarded. And the application of exosomes in clinical treatment of cancers is a challenging but intriguing endeavor that needs further investigation and exploration.

## 6. Conclusion and Future Prospective

Increasing evidence concerning exosomes has stressed their important roles in cancer development and clinical application recently. Exosome-derived cargos are involved in the pathophysiological processes of cancers, including cancer development, migration, drug resistance, and metastasis. In this review, we have elaborated upon the characteristics, biogenesis, isolation, purification, and identification of exosomes and summarized the roles of exosomal RNAs (miRNAs, lncRNAs, and circRNAs) and proteins in cancers. Investigation of the molecular mechanisms underlying the biogenesis and biological functions of exosomes will help to design more novel therapeutic methods targeting exosome-mediated pathophysiological processes in cancers.

It is noteworthy that various exosomal molecules are applied for developing emerging diagnostic strategies and clinical treatments of cancers. Obviously, the specific RNA in exosomes is one of the most robust surrogates of cancers and affects the current states of cancers. Thus, the corresponding targeted agents for these RNAs and molecules can be designed, which can be further personalized medicine. In addition to the research progress in mechanism, there are also further challenges in clinical application. The current knowledge on the roles of exosomes in cancers is limited; therefore, their potentials in the diagnosis and treatment of cancers require extensively studied.

Although exosome-based cancer treatment exerts great potentials, there are still many problems need to be solved until clinical application. The technologies of production and quality control are flawed. Although some preclinical studies evaluate the roles of exosomes as therapeutic drug delivery carriers in the cancer therapy and suggest their clinical application potentials, few exosomes-based clinical trials have been conducted, which might be the focus in the future research. Besides, several technical limitations, including high quality and bulk exosome preparation based on standard protocol, and exosome target specificity, are the biggest barriers presently impeded exosome-based diagnostic and therapeutic applications. Currently, there is still a long way to overcome the difficulty in the exosome-based therapeutic strategies in the treatment of cancers. Exosomes-based research might need to put more energy into in vivo models and clinical application in the future so that it is helpful to elucidate these questions.

## Figures and Tables

**Table 1:** The roles of exosomal miRNAs in cancers.

Cancer type	Exosomal miRNAs	Functions of exosomal miRNAs	References
BCa	miR-10b	Associated with the acquisition of malignant characteristics	[28]
	miR-223	Promote the invasion of breast cancer cells	[29]
	miR-19a	Represent a biomarker	[30]
	miR-105	A potent regulator of migration	[31]
	miR-379	Tumor suppressor	[32]
	miR-222	Upregulated in BCa patients with lymphatic metastasis	[33]
	miR-770	Inhibit the chemoresistance and metastasis	[34]
	miR-181d-5p	Promote epithelial-mesenchymal transition	[35]
Bladder cancer	miR-23b	Acquire metastatic potentials	[36]
Colon cancer	miR-193a	Inhibit cell proliferation and cause cell cycle G1 arrest	[37]

CRC	Let-7a, miR-1229, miR-1246, miR-150, miR-21, miR-223, and miR-23a	Upregulated in serum exosomes of primary CRC patients	[38]
	miR-301a and miR-23a	Upregulated in serum samples of CRC patients	[39]
	miR-17-5p and miR-92a-3p	Prognostic biomarker	[40]
	miR-150-5p	Diagnosis and prognosis	[41]
	miR-27a and miR-130a	Diagnosis and prognosis	[42]
	miR-196b-5p	Diagnosis and prognosis	[43]
	miR-25-3p	Promote premetastatic niche formation	[44]
ESCC	miR-21	Upregulated in serum from ESCC patients	[45]
GBM	miR-21	Upregulated in GBM patients	[46]
Glioma	miR-146b	Inhibit glioma growth	[47]

HCC	miR-1247-3p	Correlated with lung metastasis in HCC patients	[48]
	miR-122	Increased the antitumor efficacy of sorafenib on HCC	[25]
	miR-101, miR-106b, miR-122, and miR-195	Downregulated in the serum exosome of HCC patients	[49]
	miR-718	Inhibit cell proliferation of HCC cells	[50]
	miR-335-5p	Inhibit HCC cell proliferation and invasion, induce HCC tumor shrinkage	[51]
Leukemia	miR-210	Upregulated in exosomes and enhances endothelial migration and tube formation	[52]

Lung cancer	miR-29a	Tumor growth and metastasis	[53]
	miR-17-3p, miR-21, miR-106a, miR-146, miR-155, miR-191, miR-192, miR-203, miR-205, miR-210, miR-212, and miR-214	Diagnostic markers	[54]
	miR-155 and low let-7a-2	Diagnosis and prognosis	[55]
Lymphoma	miR-155, miR-210, and miR-21	Diagnostic markers	[56]
MM	miR-135b	Upregulated in exosomes from HR-MM and enhances endothelial tube formation	[57]

Ovarian cancer	miR-30a-5p	Promote malignant phenotypes of ovarian cancer	[58]
	miR-21, miR-141, miR-200a, miR-200c, miR-200b, miR-203, miR-205, and miR-214	Surrogate diagnostic markers for biopsy profiling	[59]
	miR-21	Promote oncogenic transformation in target cells	[60]
PaCa	miR-4306, miR-4644, miR-3976, and miR-1246	Upregulated in serum exosomes of PaCa patients	[61]

PC	miR-125b, miR-130b, and miR-155	Promote neoplastic transformation in adipose derived stem cells	[62]
	miR-196a-5p and miR-501-3p	Downregulated in exosomes from PC patients	[63]
	miR-141, miR-375, miR-107 and miR-574-3p	Biomarkers for the diagnosis, staging, and prediction of PC	[64]
	miR-1290 and miR-375	Prognostic biomarkers for castration-resistant PC patients	[65]
PDAC	miR-10b	Upregulated in plasma-derived exosomes from PDAC patients	[66]
	miR-181a, miR-10 b, miR-21, and miR-30c	Upregulated in exosomes from PDAC patients	[67]
	miR-145-5p	Inhibit the growth of xenograft tumors	[68]

BCa, breast cancer; CRC, colorectal cancer; ESCC, esophageal squamous cell cancer; GBM, glioblastoma; HCC, hepatocellular carcinoma; MM, multiple myeloma; PaCa, pancreatic cancer; PC, prostate cancer; PDAC, pancreatic ductal adenocarcinoma.

**Table 2:** The roles of exosomal lncRNAs in cancers.

Cancer type	Exosomal lncRNAs	Functions of exosomal lncRNAs	References
BCa	lncRNA-SNHG1	Promote trastuzumab chemoresistance	[73]
	lncRNA UCA1	Induce drug resistance	[74]
	lncRNA AGAP2-AS1	Induce drug resistance	[75]
	lncRNA AFAP1-AS1	Induce drug resistance	[76]
	lncRNA H19	Induce drug resistance	[77]

CRC	LNCV6_116109, LNCV6_98390, LNCV6_38772, LNCV_108266, LNCV6_84003, and LNCV6_98602	Upregulated in CRC patients	[78]
	lncRNA LINC02418	Diagnosis and prognosis	[79]
	lncRNA RPPH1	Diagnostic marker	[80]
	lncRNA CRNDE-h	Diagnosis and prognosis	[81]
GC	LncRNA HOTTIP	Upregulated in serum exosomes from GC patients	[82]
HCC	LncRNA-H19	Promote angiogenesis and adhesion of CD90^+^Huh7 cells to endothelial cell monolayer	[83]
NSCLC	lncRNA MALAT-1	Promote cell proliferation and migration	[84]
RCC	LncRNA-ARSR	Transmit to sensitive cells and disseminate sunitinib resistance	[85]

BCa, breast cancer; CRC, colorectal cancer; GC, gastric cancer; HCC, hepatocellular carcinoma; NSCLC, non-small-cell lung cancer; RCC, renal cell carcinoma.

**Table 3:** The roles of exosomal circRNAs in cancers.

Cancer type	Exosomal circRNAs	Functions of exosomal circRNAs in cancers	References
CRC	circ-ABCC1	Promote CRC progression	[87]
	circ-KLDHC10	Upregulated in serum of CRC patients	[88]
	circ-0004771	Diagnosis and prognosis	[89]
GC	ciRS-133	Promote cancer cachexia, oxygen consumption, and heat production	[90]
	circ-SHKBP1	Promote GC progression	[91]
	circ-RanGAP1	Promote invasion and metastasis	[92]
	circ-0032821	Promote tumor growth	[93]
Glioma	circ-MMP1	Promote the progression of glioma	[94]
HCC	circ-ZNF652	Promote cell proliferation, migration, invasion, and glycolysis	[95]
	circ-100284	Promote the cell cycle	[96]
	circ-DB	Promote tumor cell proliferation	[97]
	circ-100338	Promote HCC metastasis	[98]
	circ- Cdr1as	Promote HCC proliferation and migration	[99]
	circ-0051443	Downregulated in plasma exosomes and tissues from HCC patients	[100]
	circ-PTGR1	Upregulated in serum exosomes from HCC patients	[101]
	circ-UHRF1	Upregulated in human HCC tissues	[102]
	circ-AKT3	Upregulated in exosome from HCC patients	[103]
LSCC	circRASSF2	Promote cancer cell proliferation and migration	[104]
LUAD	circ-002178	Upregulated in the LUAD tissues and LUAD cancer cells	[105]
MM	circ-MYC	Upregulated in exosome from MM patients	[106]
OSCC	circGDI2	Inhibit OSCC cell proliferation, migration, invasion, and glycolysis	[107]
PC	circ-0044516	Promote cell proliferation and metastasis	[108]
PDAC	circ-PDE8A	Promote the invasive growth of PDAC cell	[109]
	circ-IRAS	Promote tumor invasion and metastasis	[110]
UCB	circ-PRMT5	Upregulated in serum and urine exosomes from UCB patients	[111]

CRC, colorectal cancer; HCC, hepatocellular carcinoma; LSCC, laryngeal squamous cell carcinoma; LUAD, lung adenocarcinoma; MM, multiple myeloma; OSCC, oral squamous cell carcinoma; PC, prostate cancer; PDAC, pancreatic ductal adenocarcinoma; UCB, urothelial carcinoma of the bladder.

**Table 4:** The roles of exosomal proteins in cancers.

Cancer type	Exosomal proteins	Functions of exosomal proteins	References
BC	L-plastin	Promote osteolysis	[116]

CRC	CD147	Diagnosis and prognosis	[117]
	IRF-2	Remodel the lymphatic network	[118]

GBM	IL-6, IL-8, and angiogenin	Diagnosis and prognosis	[119]
	EGFR, EGFRvIII, and TGF-*β*	Diagnosis	[120]
	EGFR, EGFRvIII, PDPN, and IDH1 R132H	Diagnosis and prognosis	[121]
	VEGF-A	Diagnosis	[122]
	CD97	Promote metastasis	[123]
	HMGB1	Diagnosis	[124]
Glioma	IL13QD	Diagnosis and prognosis	[125]

Lung cancer	IQGAP, MUC5B, BPIFA1, and CRNN	Diagnosis	[126]
	LRG1	Diagnosis	[127]

Melanoma	HSP70, HSP90, TYRP2, VLA-4, and MET	Diagnosis and prognosis	[128]
	CD63 and Caveolin-1	Diagnosis	[129]
	MHC-I/peptide complexes	Diagnosis	[130]
	Podoplanin	Diagnosis	[131]
	TYRP2, VLA-4, HSP70, and HSP90	Diagnosis and prognosis	[128]
Myeloma	HSP70	Diagnosis	[132]
NSCLC	Amphiregulin	Promote osteoclastogenesis and metastasis	[133]

Ovarian cancer	HNRHPU, U2AF2 TGM2, and U2AF1	Diagnosis	[134]
	EpCAM and CD24	Diagnosis	[135]
PaCa	Glypican-1	Diagnosis	[136]

PC	PDCD6IP, FASN, XPO1, and ENO1	Diagnosis	[137]
	*α*v*β*6 integrin	Regulate monocyte M2 polarization	[138]

PDAC	MIF	Promote fibronectin secretion and metastasis	[139]
	CD151 and TSPAN8	Promote EMT gene expression	[140]

BC, breast cancer; CRC, colorectal cancer; GBM, glioblastoma; GC, gastric cancer; NSCLC, non-small-cell lung cancer; PaCa, pancreatic cancer; PC, prostate cancer; PDAC, pancreatic ductal adenocarcinoma.
